# Supplementary figures and images for: A machine learning framework for the prediction of chromatin folding in Drosophila using epigenetic features
Source: PeerJ Comput Sci. 2020 Nov 30;6:e307. doi: 10.7717/peerj-cs.307 (PMC7924456; doi:10.7717/peerj-cs.307)

**A** Schneider-2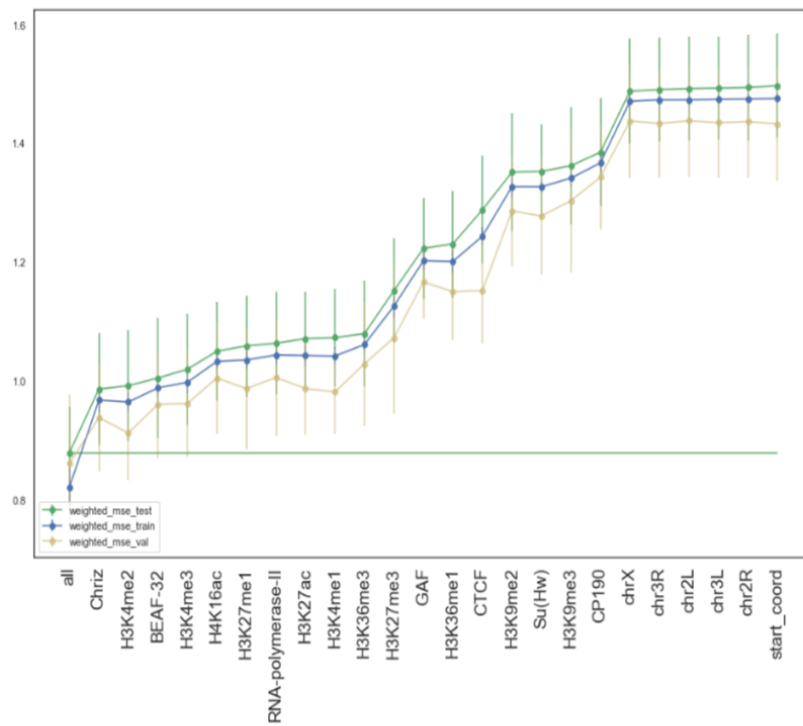**B** Kc167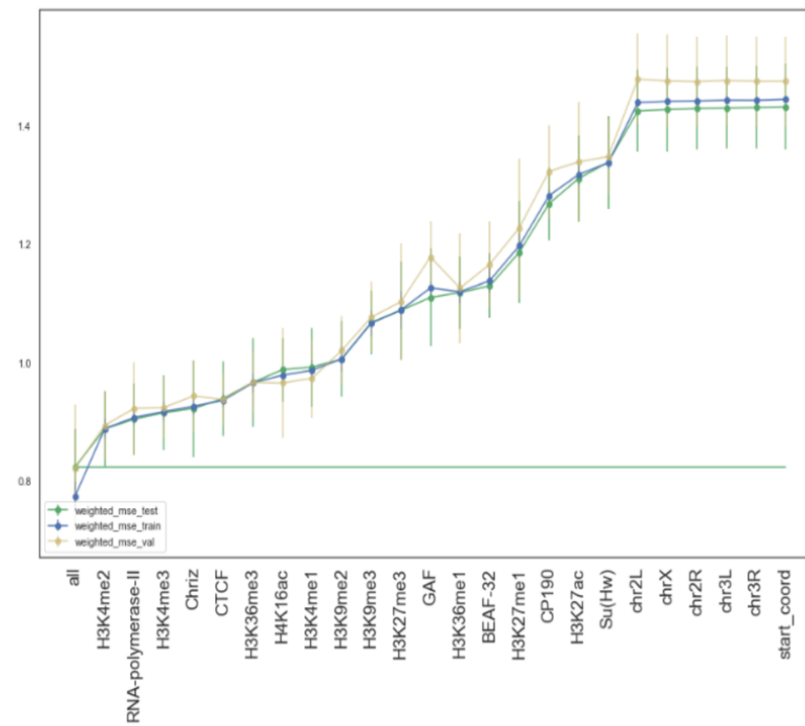**C** DmBG3-c2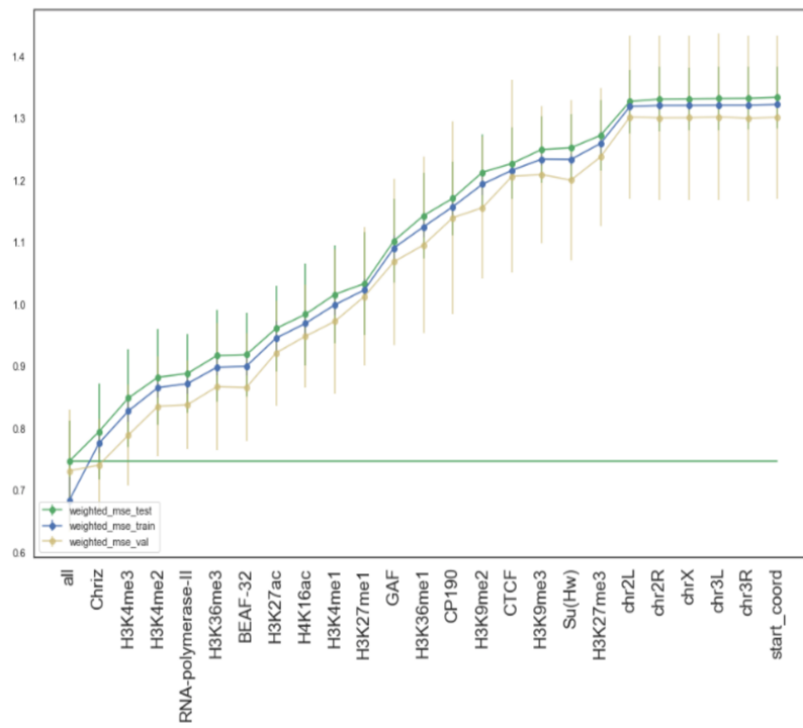**D** All together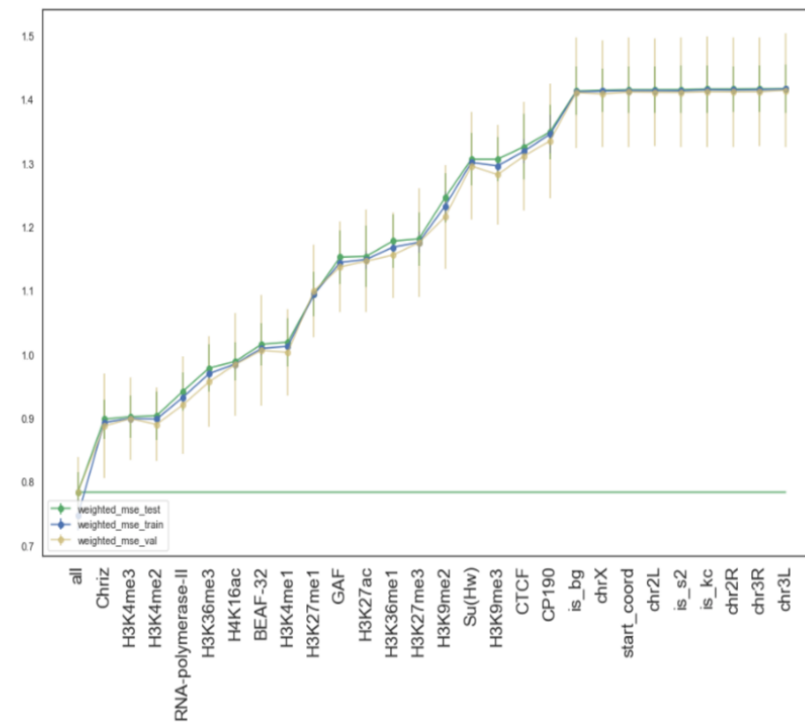

Supplement: Supplemental Information 1 — Results of biLSTM RNN using (A) Schneider-2, (B) Kc167, (C) DmBG3-c2 and (D) all three cell lines together. The green lines reflect the weighted MSE scores on the test sets, the blue lines show the wMSE on the train sets and the yellow lines correspond to the same metric on the validation datasets. [file peerj-cs-06-307-s001.pdf]

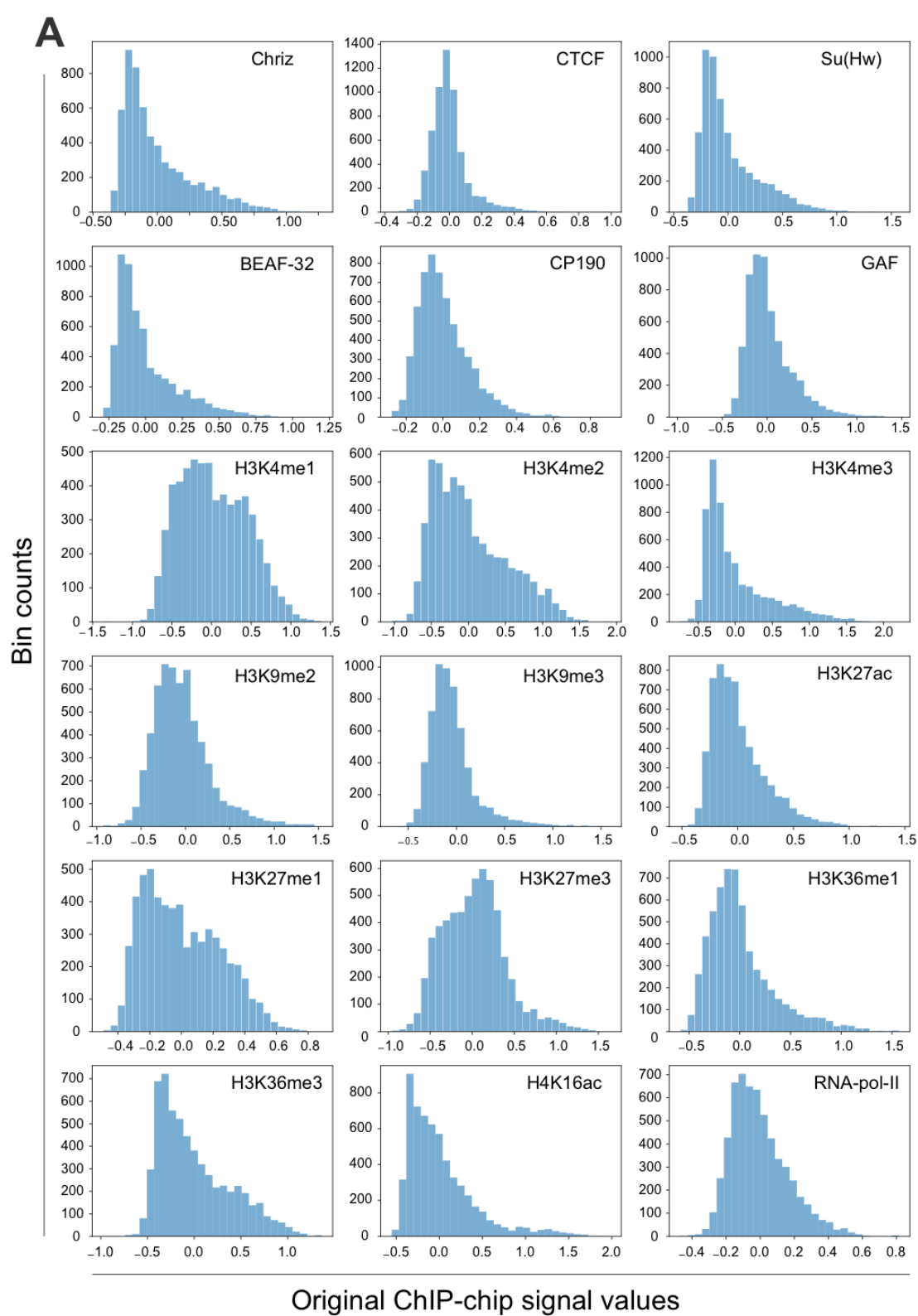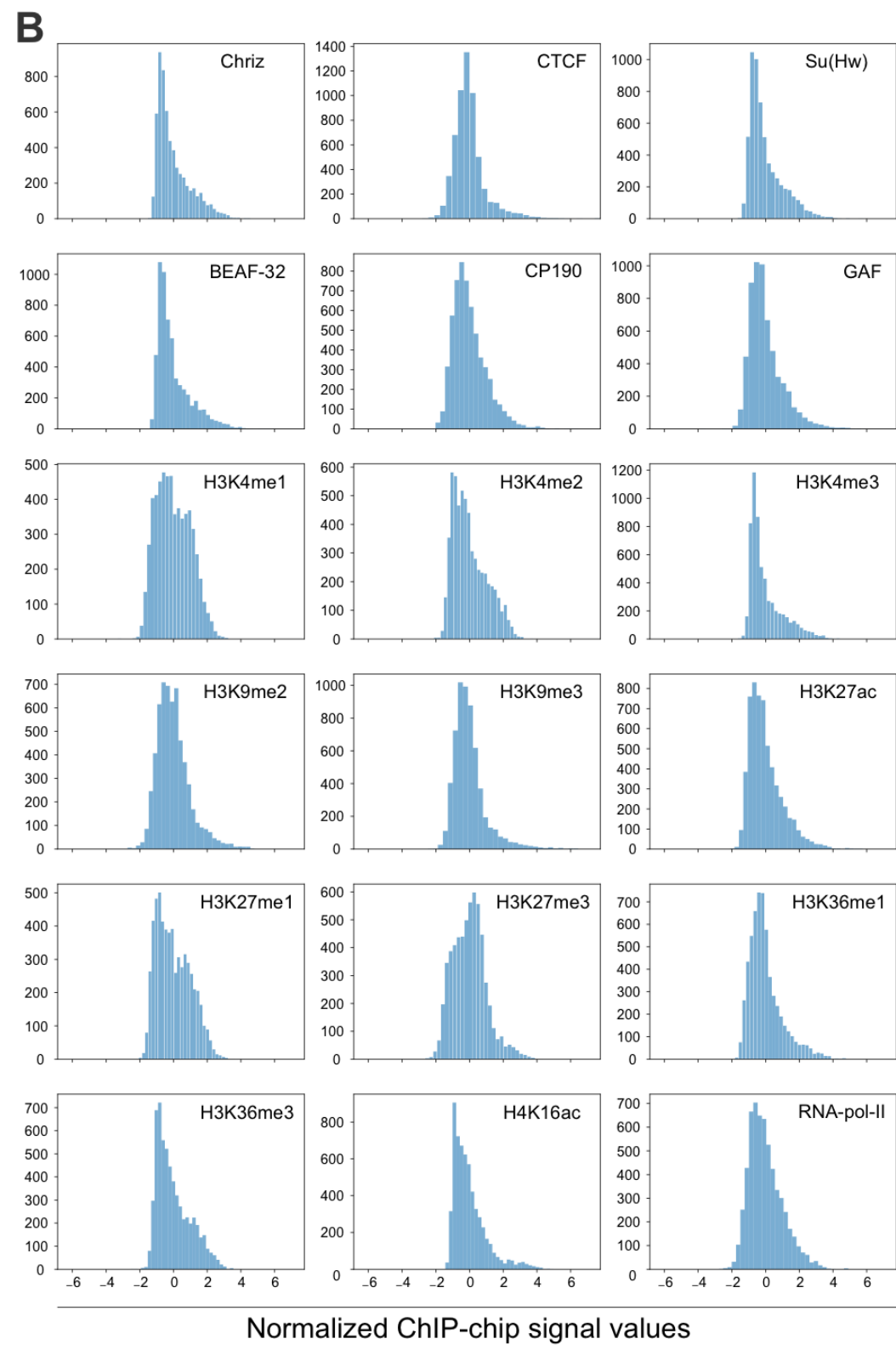

Supplement: Supplemental Information 2 — Each histogram corresponds to the distribution of the analysed ChIP-chip features. Before the normalization (A), the distributions are not centered at zeros and have varying variance. After normalization (B), all the features are rescaled to the same mean and variance. [file peerj-cs-06-307-s002.pdf]
