# Supplementary material for: A machine learning framework for the prediction of chromatin folding in Drosophila using epigenetic features
Source: PeerJ Comput Sci. 2020 Nov 30;6:e307. doi: 10.7717/peerj-cs.307 (PMC7924456; doi:10.7717/peerj-cs.307)
Supplement: Supplemental Information 3 — Each number in the table corresponds to the modENCODE ID. The columns identify the Drosophila cell lines. The rows show the chromatin factors. [file peerj-cs-06-307-s003.pdf]

Table 1. The modENCODE IDs of chromatin factors for three selected *Drosophila* cell lines.

	modEncode IDs		
name	Schneider-2	Kc167	DmBG3-c2
Chriz	279	277	275
CTCF	3749	3749	3671
Su(Hw)	5147	3801	3717
BEAF-32	922	3745	3663
CP190	925	3748	3666
GAF	3753	3753	2651
H3K4me1	3760	5138	2653
H3K4me2	965	4935	2654
H3K4me3	3761	5141	967
H3K9me2	311	938	310
H3K9me3	4183	3013	312
H3K27ac	3757	3757	295
H3K27me1	3943	3942	3941
H3K27me3	298	5136	297
H3K36me1	3170	3003	299
H3K36me3	303	302	301
H4K16ac	320	318	316
RNA-polymerase-II	329	328	950
